# Rio Negro Virus Infection, Bolivia, 2021

**DOI:** 10.3201/eid2908.221885

**Published:** 2023-08

**Authors:** Roxana Loayza Mafayle, Maria E. Morales-Betoulle, Shannon Whitmer, Caitlin Cossaboom, Jimmy Revollo, Nelly Mendoza Loayza, Hilary Aguilera Méndez, Joel Alejandro Chuquimia Valdez, Freddy Armijo Subieta, Maya Xochitl Espinoza Morales, María Valeria Canedo Sánchez, Miriam Eugenia Romero Romero, Aaron C. Brault, Holly R. Hugues, Jairo Mendez-Rico, Jason H. Malenfant, Trevor Shoemaker, John D. Klena, Joel M. Montgomery, Jhonatan David Marquina Salas

**Affiliations:** Centro Nacional de Enfermedades Tropicales, Santa Cruz de la Sierra, Bolivia (R. Loayza Mafayle, J. Revollo, N. Mendoza Loayza, H. Aguilera Méndez, J.A. Chuquimia Valdez, J.D. Marquina Salas);; Centers for Disease Control and Prevention, Atlanta, Georgia, USA (M.E. Morales-Betoulle, S. Whitmer, C. Cossaboom, J.H. Malenfant, T. Shoemaker, J.D. Klena, J.M. Montgomery);; Unidad de Epidemiología, Ministerio de Salud, La Paz, Bolivia (F. Armijo Subieta);; Unidad de Gestión de Riesgos en Salud Ambiental, Emergencias y Desastres Ministerio de Salud, La Paz (M.X. Espinoza Morales);; Bolivia Ministerio de Salud Programa Salud Familiar Comunitaria Intercultural, Padcaya, Bolivia (M.V. Canedo Sánchez, M.E. Romero Romero);; Centers for Disease Control and Prevention, Fort Collins, Colorado, USA (A.C. Brault, H.R. Hugues);; Pan-American Health Organization, Washington, DC, USA (J. Mendez-Rico)

**Keywords:** Rio Negro virus, zoonoses, viruses, arboviruses, vector-borne infections, febrile infections, Bolivia

## Abstract

In May 2021, an agricultural worker originally from Trementinal, Argentina, sought treatment for febrile illness in Tarija, Bolivia, where he resided at the time of illness onset. The patient tested negative for hantavirus RNA, but next-generation sequencing of a serum sample yielded a complete genome for Rio Negro virus.

Rio Negro virus (RNV; family Togaviridae, genus *Alphavirus*), a Venezuelan equine encephalitis virus (VEEV) antigenic subtype VI virus, was first reported in 1987 after being isolated from mosquitoes collected in Chaco, Argentina (*1*). The virus has since been isolated or molecularly detected in mosquitoes and rodents in Argentina and bats in Uruguay (*2**–**6*). Although RNV was serologically associated with an outbreak of undifferentiated febrile illness in Argentina, molecular evidence of RNV infection in humans is lacking (*4*,*7*,*8*). High RNV seroprevalence among horses in Uruguay suggests the virus likely circulates throughout the region (*9*). Dengue viruses 1–4 are leading causes of acute febrile illnesses in Latin America, but confirmatory testing is often not performed. Surveillance is also not routinely performed for other viral etiologies of acute febrile illnesses (e.g., arenaviruses, hantaviruses, other arboviruses). In regions of Bolivia where hantaviruses are known to circulate, a national surveillance program collects blood samples, along with clinical and epidemiologic information, including risk factors associated with hantavirus infection (e.g., agricultural work) from patients manifesting nonspecific signs and symptoms (e.g., fever, headache, nausea, myalgia, arthralgia, shortness of breath) for hantavirus testing at the Centro Nacional de Enfermedades Tropicales (CENETROP) in Santa Cruz de la Sierra, Bolivia. After hantavirus testing, a subset of RNA specimens was sent to the US Centers for Disease Control and Prevention (CDC) for further characterization using next-generation sequencing (NGS). We report molecular evidence of human infection with RNV, characterized by NGS and genomic analysis. 

A 21-year-old man, a migrant agricultural worker originally from Trementinal, Argentina, with no related medical history, sought treatment on May 31, 2021, in Padcaya Municipality, Tarija Department, Bolivia, where he resided at the time of illness onset (Appendix Figure). On arrival, he reported a 1-day history of fever, chills, headache, nausea, arthralgia, myalgia, thoracic pain, back pain, and hyperemia; his temperature at the time, 37.6°C, was the maximum during his hospitalization. Physical examination revealed bibasilar crackles but observations were otherwise unremarkable. He was admitted to hospital with suspected SARS-CoV-2 or hantavirus infection, but other infectious etiologies, such as dengue or a bacterial urinary tract infection, were also considered. The antimicrobial levofloxacin and corticosteroids dexamethasone and betamethasone were empirically prescribed. Initial clinical testing included complete blood count, basic metabolic panel, and urinalysis; all results were unremarkable. Results of a SARS-CoV-2 rapid test was negative. A blood sample was sent to CENETROP for hantavirus testing. The patient ultimately made a full recovery and was discharged after 5 days on June 5, 2021. 

In December 2021, archived samples collected for hantavirus surveillance were inactivated in CENETROP’s Biosafety Level 3 laboratory and tested for hantavirus antibodies and RNA using CDC ELISA and real-time reverse transcription PCR (RT-PCR) (Appendix). RNA from RT-PCR–positive and –negative samples was sent to CDC for NGS. The specimen collected from the patient was negative for hantavirus RNA. A near-complete genome (excluding 40 of >11K nt) was obtained using NGS and identified by genomic analysis as RNV (Figure), a result supported by alphavirus RT-PCR followed by sequencing. The RT-PCR amplicon had highest identity, 97.8% (441/451 nt), to GenBank RNV reference strain NC_038674, suggesting the amplicon was not a product of laboratory contamination. RNV was not identified in a subset of hantavirus RNA–positive (n = 7) and –negative (n = 4) specimens collected from the same area or in negative controls.

**Figure:**
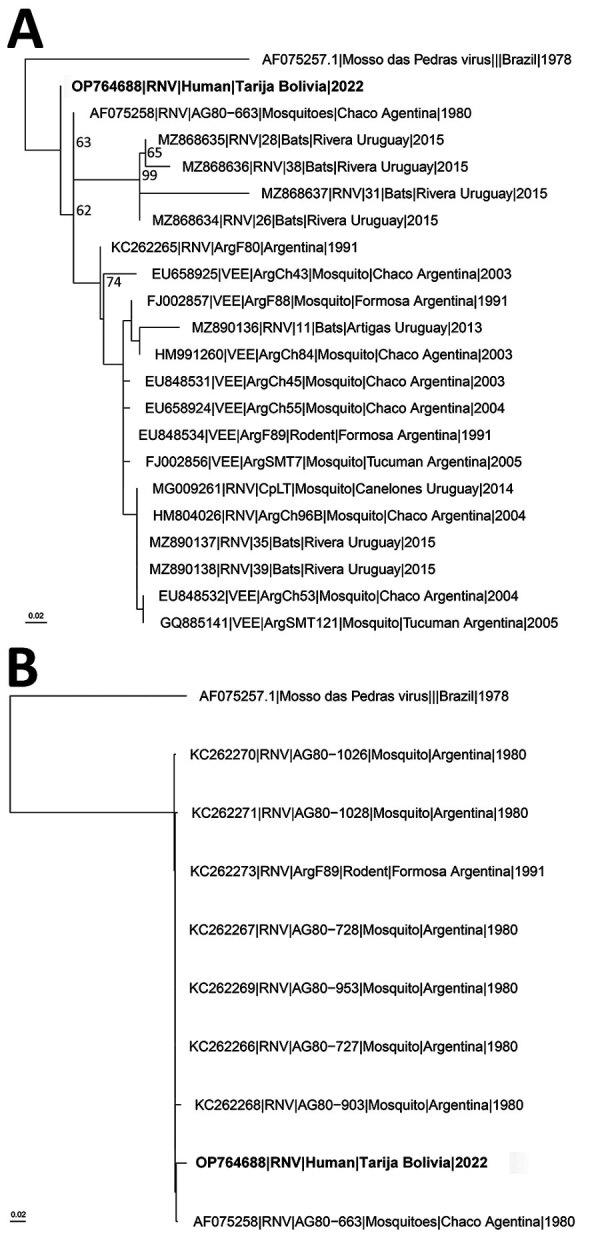
Inferred phylogenetic relationships of Rio Negro viruses collected from mosquitoes, bats, and rodents in South America and the human-derived RNV sequence described in this report from Tarija, Bolivia (bold text). A) Phylogenetic tree made with an alignment of partial nsP4 sequences of Rio Negro viruses. Mossa das Pedras virus is included as an outgroup. B) Phylogenetic tree made with an alignment of partial E3/GP2 sequences of Rio Negro viruses. Mossa das Pedras virus is included as an outgroup. GenBank accession numbers are provided. Scale bars indicate substitutions per site.

VEEV is a substantial human and animal pathogen considered a persistent zoonosis in Latin America; RNV is a VEEV subtype VI arbovirus, closely related to subtype I viruses, which have resulted in large-scale human outbreaks of >100,000 cases (*10*). RNV has been isolated from *Culex* spp. mosquitoes and rodents in Argentina (*2**–**5*). Evidence of RNV infection in humans has been limited to serologic studies (*4*,*7*,*8*). We report molecular evidence of human infection with RNV in a patient who sought treatment with signs and symptoms of a nonspecific febrile illness. No serum specimen from the patient was available for serologic testing for RNV. No other complete or nearly complete pathogen genomes (>50% coverage) were generated by de novo analysis in this patient sample, and RNV RNA was not detected in other tested specimens or the negative sequencing control. 

Because the patient sought treatment in a rural area of Bolivia, follow-up has been challenging, and limited information is available on the patient’s epidemiologic history. Additional information is lacking on the patient’s travel history and potential exposures to mosquitoes, rodents, bats, and horses that could further characterize the potential distribution and risk factors for RNV infection in the region. The true burden of RNV as a cause of human disease in Bolivia and the region is unknown; however, because initial manifestation consists of nonspecific signs and symptoms, RNV infections could be overlooked or misdiagnosed. To bolster surveillance and diagnostic capacity for RNV and other emerging viruses, it is critical for healthcare sectors in Latin America to look beyond dengue and other common causes of acute febrile illnesses. 

Appendix Additional information about a case of Rio Negro virus infection in a person in Bolivia.
