# Supplementary material for: Rio Negro Virus Infection, Bolivia, 2021
Source: Emerg Infect Dis. 2023 Aug;29(8):1705–8. doi: 10.3201/eid2908.221885 (PMC10370837; doi:10.3201/eid2908.221885)
Supplement: Appendix — Additional information about a case of Rio Negro virus infection in a person in Bolivia. [file 22-1885-Techapp-s1.pdf]

Article DOI: https://doi.org/10.3201/eid2908.221885

EID cannot ensure accessibility for supplementary materials supplied by authors. Readers who have difficulty accessing supplementary content should contact the authors for assistance.

Rio Negro Virus Infection, Bolivia, 2021

Appendix

Supplemental Materials and Methods

Sample Inactivation and RNA Extraction

Different types of specimens (blood, serum) were collected from cases meeting the case definition used in Bolivia for suspected hantavirus. Prior to serology testing or RNA extraction, specimens were inactivated in the biosafety level 3 (BSL3) at CENETROP following CDC's biosafety guidelines (https://www.cdc.gov/safelabs/resources-tools/biosafety-resources-and-tools.html). In short, specimens were inactivated in the BSL3 laboratory at CENTROP, with enhanced personal protective equipment for aerosol precautions, like N95 masks or powered air purifying respirators (PAPRs). For serology, 42 µL of each specimen were inactivated in 1 mL of a buffer containing 2 detergents, followed by incubation at 56°C and tested as previously described using the CDC's internally developed SNV hantavirus IgM, IgG ELISA assays (*1*,*2*). For RNA extraction, 140 µL of each specimen was placed in 560 µL of lysis buffer (AVL); after 10 min, 560 µL of ethanol were added; 10 min incubation completed the inactivation. Tubes were sealed and decontaminated prior to transfer from BSL3 to BSL2 where RNA extraction was performed with the QIAGEN QIAamp Viral RNA Mini Kit (https://www.qiagen.com) following manufacturer's instructions. RNA samples were tested using a CDC RT-qPCR assay to detect South American hantaviruses (manuscript in preparation). Positive and negative RNA samples were sent to CDC for further characterization by Next Generation Sequencing (NGS) and phylogenetic analysis.

Next-Generation Sequencing

Extracted RNA was treated with Roche RNase-free DNase (https://www.sigmaaldrich.com) and NEBNext Ultra II Directional RNA library preparation kit (New England Biolabs;

https://www.neb.com). Libraries were sequenced using either an Illumina MiSeq or MiniSeq high output (2 × 150 cycles) system (https://www.illumina.com). Pathogen discovery was conducted by de novo assembling reads with SPAdes (-k auto, v3.14.0) and contigs were blasted against the Genbank nonredundant database to identify the most closely related reference sequences. De novo assembly generated a complete RNV full length genome. To confirm the de novo assembly, reads were mapped to the full length RNV contig and RNV reference genome (AF075258) using the Geneious medium-low sensitivity/fast, iterate 2 times mapper (https://www.geneious.com). The consensus genomes were identical to the full-length de novo assembled RNV contig. Evolutionary history was inferred using all available RNV partial genomes from GenBank using raxml (-m GTRGAMMA -p $RANDOM -f a -x $RANDOM -N 1000) with bootstrap support provided by 1,000 iterations. RNV genomes were deposited to GenBank: OP764688.

Confirmatory Alphavirus RT-PCR and Sequencing

All methods followed manufacturer protocols unless otherwise stated. RNV was amplified from extracted RNA using alphavirus consensus primers previously described (*3*) and the One-step RT-PCR kit (QIAGEN) utilizing 10µL of RNA in a reaction volume of 50µL. RNV was amplified on a BioRad C1000 thermal cycler (https://www.bio-rad.com) using 50°C annealing temperature and 45 cycles. Positive amplification was visualized on a Thermo Fisher 1.5% SyberSafe E-Gel (https://www.thermofisher.com). RT-PCR reactions were cleaned up using the Qiaquick PCR purification mini kit (QIAGEN) and capillary sequenced on an ABI 3500xL Genetic Analyzer (Thermo Fisher), using the forward and reverse primers from RT-PCR amplification with the BigDye termination kit (Thermo Fisher). The resulting 453bp sequence was confirmed RNV using BLAST analysis (https://blast.ncbi.nlm.nih.gov/Blast.cgi).

Acknowledgments

All the authors supported the investigations described herein, decided to publish the manuscript, and critically reviewed the manuscript. The first four authors collected and analyzed the data. A subgroup of the authors initially drafted the manuscript. The senior authors vouch for the accuracy and completeness of the data. There was no commercial funding for this study. Details of author contributions can be found below.

Conceptualization: R.L.M., M.M.B., S.M.W.,C.M.C., J.R., N.M., J.M.R., , T.S., J.D.K., J.M.M. and J.M. S. Formal analysis: R.L.M., M.M.B., S.M.W., C.M.C., J.R., H.A.M., J.A.C.V., J. M. H.H., and A.B. Investigation: R.L.M., M.M.B., S.M.W., C.M.C., J.R., N.M., J.M.R., J.M., N.F.A.S, M.X.E.M., T.S., J.D.K., J.M.M., and J.D.M.S.

Methodology: R.L.M., M.M.B., S.M.W., C.M.C, J.R., H.H., and A.B. Patient care and clinical data collection: M.V.C.S., and M.E.R.R. Visualization: S.M.W., C.M.C. Funding acquisition: J.D.M.S, N.M., N.F.A.S, M.X.E.M., J.M.R., T.S., J.D.K., and J.M.M. Project administration: R.L.M., M.M.B., S.M.W., C.M.C., J.M.R., T.S., J.D.K., J.M.M. and J.D.M.S. Supervision: N.M., J.M.R., T.S., J.D.K., J.M.M. and J.D.M.S, Writing, original draft: M.M.B., S.M.W., and, C.M.C. Writing, review, and editing: R.L.M., S.M.W., M.M.B., C.M.C., J.R., J.M., A.B., H.H., T. S., J.D.K., and J.M.M.

References

1. Loayza Mafayle R, Morales-Betoulle ME, Romero C, Cossaboom CM, Whitmer S, Alvarez Aguilera CE, et al. Chapare hemorrhagic fever and virus detection in rodents in Bolivia in 2019. N Engl J Med. 2022;386:2283–94.
2. MacNeil A, Comer JA, Ksiazek TG, Rollin PE. Sin Nombre virus-specific immunoglobulin M and G kinetics in hantavirus pulmonary syndrome and the role played by serologic responses in predicting disease outcome. J Infect Dis. 2010;202:242–6.
3. Sánchez-Seco MP, Rosario D, Quiroz E, Guzmán G, Tenorio A. A generic nested-RT-PCR followed by sequencing for detection and identification of members of the alphavirus genus. J Virol Methods. 2001;95:153–61.

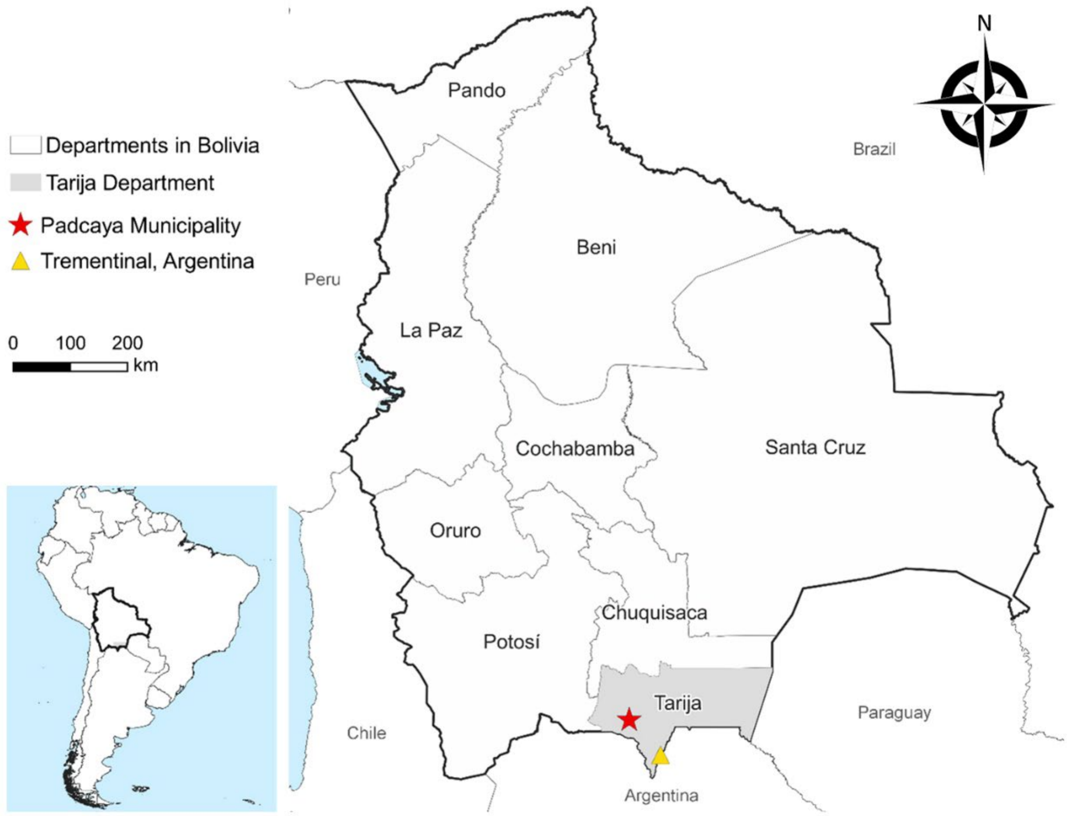

Appendix Figure. Map depicting Trementinal, Argentina, where the patient was originally from, and Padcaya Municipality, Tarija Department, Bolivia, where the patient first developed symptoms and was admitted for care.
